# Visual modulation of auditory evoked potentials in the cat

**DOI:** 10.1038/s41598-024-57075-1

**Published:** 2024-03-26

**Authors:** Xiaohan Bao, Stephen G. Lomber

**Affiliations:** 1 Integrated Program in Neuroscience, McGill University, Montreal, QC H3G 1Y6 Canada; 2 Department of Physiology, McGill University, McIntyre Medical Sciences Building, Rm 1223, 3655 Promenade Sir William Osler, Montreal, QC H3G 1Y6 Canada

**Keywords:** Auditory system, Sensory processing

## Abstract

Visual modulation of the auditory system is not only a neural substrate for multisensory processing, but also serves as a backup input underlying cross-modal plasticity in deaf individuals. Event-related potential (ERP) studies in humans have provided evidence of a multiple-stage audiovisual interactions, ranging from tens to hundreds of milliseconds after the presentation of stimuli. However, it is still unknown if the temporal course of visual modulation in the auditory ERPs can be characterized in animal models. EEG signals were recorded in sedated cats from subdermal needle electrodes. The auditory stimuli (clicks) and visual stimuli (flashes) were timed by two independent Poison processes and were presented either simultaneously or alone. The visual-only ERPs were subtracted from audiovisual ERPs before being compared to the auditory-only ERPs. N1 amplitude showed a trend of transiting from suppression-to-facilitation with a disruption at ~ 100-ms flash-to-click delay. We concluded that visual modulation as a function of SOA with extended range is more complex than previously characterized with short SOAs and its periodic pattern can be interpreted with “phase resetting” hypothesis.

## Introduction

It has been almost unanimously agreed that the cross-modal timing between two stimuli plays a key role in multisensory processing ^1,2^ (see Koelewijn^2^ for a review). An audiovisual disparity, or stimulus onset asynchrony (SOA), of ~ 100 ms could substantially impede the perception of simultaneity ^3–5^ and provided sufficient information for temporal order judgement ^6,7^. The improvement on the performance of perception (e.g., reaction time or accuracy) by adding stimulus from a second modality is also diminished with increasing audiovisual SOA ^8–12^. Such time sensitivity indicates that the complexity of neural circuits that are not fully understood yet is involved in audiovisual interactions, and potentially cross-modal plasticity after hearing loss.

The range of SOAs up to 100 ms, which cross-modal temporal processing (simultaneity and temporal order judgement) is sensitive to ^13–15^, has been studied in human ERP and MEG experiments ^16–22^. We refer to SOAs of this range as “short SOAs” and both types of studies have shown that short SOAs can modulate the multisensory component of ERP activities. However, longer SOAs were not extensively studied in these human ERP experiments.

Using extracellular recording or behavioral measurements, a few investigations have shed some light on the effect of long SOAs in multisensory processing. In macaque primary auditory cortex, Lakatos et al. ^23^ showed that neuronal activities evoked by a click were modulated by a preceding tactile stimulus with up to about 800-ms SOA. Fiebelkorn et al. ^24^ measured the fluctuated behavioral performance in detecting a near-threshold Gabor stimulus after a preceding tone beep up to a 6-s SOA. The findings in both studies have implied that the effect of long SOAs on multisensory interaction is due to the oscillations in the cortical excitability phase-locked to the preceding stimulus. This would be contradictory with the evoked model ^25^, where stimulus-evoked neural activity by the preceding stimulus may have a more limited effective period. Thus, we hypothesized that, in auditory ERPs, cross-modal modulation originating from a visual input should also occur with audiovisual temporal disparity beyond the range sensitive for multisensory temporal processing, where a periodic pattern of fluctuation may be observed.

**Figure 1:**
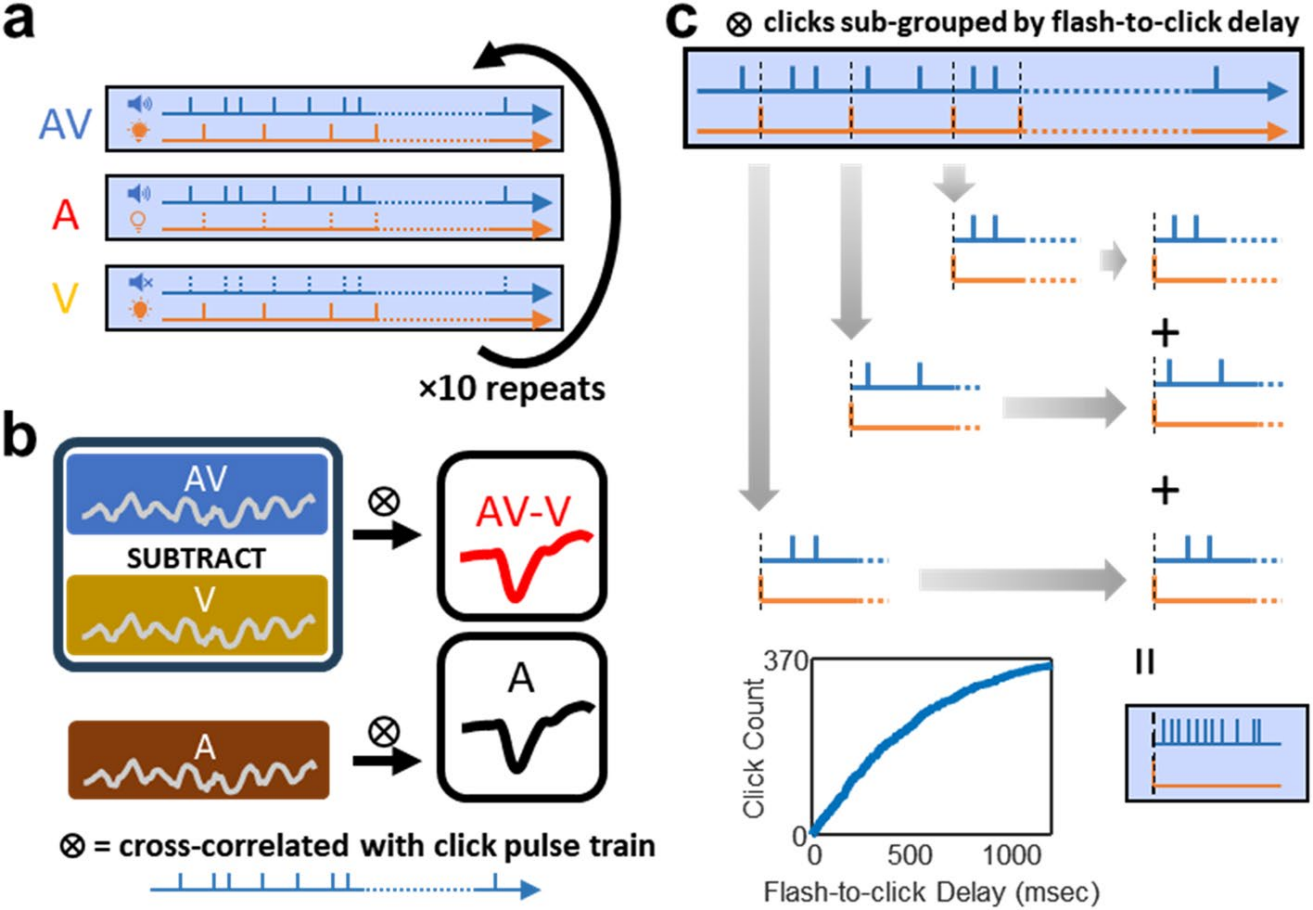
Stimulus paradigm and click grouping based on flash-to-click delays. (**a**) Three stimulus conditions, audiovisual (AV), auditory only (A) and visual only (V) were presented 10 times to each subject while EEG signal was continuously recorded. Same click train and flash train were repeatedly used in all three conditions. (**b**) EEG signal from the V condition was subtracted from AV condition in each repeat to generate an AV-V condition. For both AV-V and A conditions, epochs time-locked to click onsets were extracted and were averaged to derive cortical auditory evoked potentials (cAEPs). (**c**) For investigating the effect of audiovisual temporal disparity, clicks were sorted by flash-to-click delays and grouped into different bins. This way, cAEP waveforms can be obtained separately from different click groups. Flash-to-click delays overall spanned from 0 to about 1000 ms.

The existing ERP studies on the temporal disparity of audiovisual integration provided very limited information specific to long SOAs and its spectral patterns ^26–32^. To fill this research gap, the current study is aimed at providing unparalleled evidence of the interaction between cat ERPs in response to auditory (click) and visual (flash) stimuli and audiovisual SOAs up to 1 s (Fig. 1). We found that the amplitude of N1 from cortical auditory evoked potentials (cAEPs) in cat under dexmedetomidine sedation was affected by audiovisual SOAs. Change in N1 amplitude as a function of SOA revealed a temporal dynamic of visual modulation in an oscillatory pattern.

## Results

**Figure 2:**
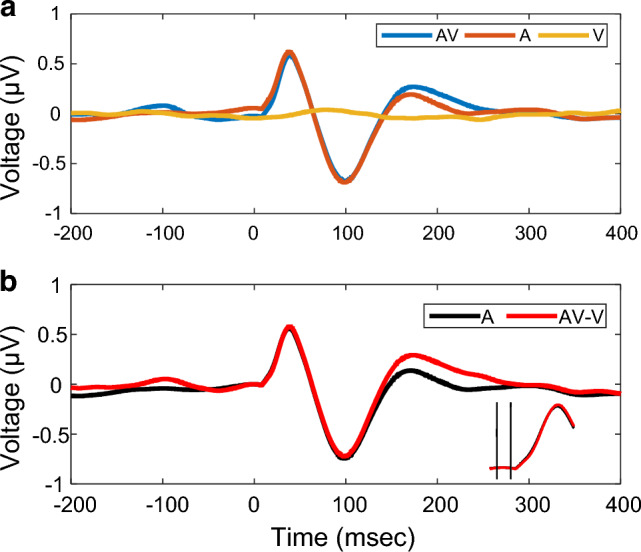
Cortical auditory evoked potentials (cAEPs) from all stimulus conditions. (**a**) Grand-averaged waveforms of cAEP in three stimuli conditions. The epochs were averaged with click onsets. Note that in the case of visual-only (V) condition, the click onsets were the same as in the auditory-only (A) and the audiovisual (AV) condition, despite that no click was presented. (**b**) Contrast of cAEP waveforms between the A and the AV-V conditions. Inset, an enlarged view of the waveform near the click onset and the baseline between the two vertical lines (from 5-ms before to 5-ms after click onsets).

Cats under dexmedetomidine sedation were presented with 1-min trains of clicks (auditory, A), flashes (visual, V), and unsynchronized clicks and flashes (audiovisual, AV) (Fig. 1a). Then, an offline bandpass filter (1–10 Hz) was applied for obtaining cortical auditory evoked potentials (cAEPs). First, we extracted epochs time-locked to click onsets from all three stimulus conditions. The grand-averaged waveforms derived from both the AV and the A conditions revealed clear cortical auditory evoked potentials (cAEPs) but not the waveform from the V condition (Fig. 2a). The flash stimuli did not seem to influence the grand-averaged waveforms of click cAEPs, due to the fact that flash and click stimuli were out of sync. This, however, may not be the case, when specific flash-to-click delays were to be investigated. Therefore, EEG signals from V condition were subtracted from the corresponding AV condition in each of the 10 repeats, generating an AV-V condition (Fig. 1b). For further data analysis, epochs were extracted from the derived AV-V and the original A conditions, respectively, for waveform averaging and peak measurements (Fig. 2b).

Data were collected from 14 cat subjects. Regardless of the flash-to-click delay, each subject was presented with 370 clicks with 10 repeats, giving rise to an average of 3700 epochs in each individual cAEP waveforms (Supplementary Fig. 1a).The cAEP waveforms from the both conditions featured a prominent positive peak component about 35-ms latency, which we referred as P1, followed by a slower and wider negative peak component at about 95-ms latency post-click, which we referred as N1 (Fig. 2b). A second positive peak component, less prominent than P1, was present at about 170-ms latency, which we referred to as P2. From the grand-average waveforms, we observed a near-perfect overlap between the AV-V and the A conditions, especially for the initial 125-ms duration after click onset, suggesting a well-preserved cAEP morphology when unsynchronized visual stimuli were simultaneously present. There appeared to be an elevation of the traces starting at 150-ms after click onset in the AV-V condition.

The P1-N1-P2 complex were observed in all subjects. The amplitudes and the latencies were measured from each of the three peak components (Table 1 and Supplementary Fig. 1a). Only P2 amplitude was significantly larger in the AV-V condition than the A condition (Δamp_*P2*_ = 0.14, *p* = 0.007 < 0.01). It was noticed in the later analysis that three subjects demonstrated more noise in their recordings. It became more apparent in peak identification, when cAEPs were analyzed in separate click groups according to the flash-to-click delay (Supplementary Fig. 1b). Excluding these three subjects, however, did not change the result of comparisons between the AV-V and the A conditions above (Δamp_*P2*_ = 0.18, *p* = 0.005 < 0.01). Although P2 amplitude demonstrated the effect of visual modulation without depending on the timing between flash and click stimuli, P1 and N1 components, as well as P2 latency, did not, which is consistent with the existing knowledge that out-of-timing visual stimulus does not affect auditory processing ^33^. To investigate how stimulus timing plays a role in the effect of visual modulation, we focused on N1 amplitude as the major measurement in the following data analysis.

**Table 1:** Amplitudes and peak times of P1-N1-P2 complex in individual subjects.

Subject no.	P1				N1				P2			
	A		AV-V		A		AV-V		A		AV-V	
	μV	ms	μV	ms	μV	ms	μV	ms	μV	ms	μV	ms
1	0.68	36	0.97	36	− 0.68	111	− 0.74	108	0.16	285	0.32	221
2	0.74	39	0.67	41	− 1.18	99	− 0.73	99	− 0.01	213	0.53	224
3	0.62	38	0.59	39	− 0.70	95	− 0.69	97	0.23	173	0.40	168
4	0.28	32	0.49	36	− 0.85	92	− 0.43	97	0.33	169	0.56	165
5	0.52	43	0.48	44	− 0.81	106	− 0.73	103	0.27	173	0.35	172
6	0.51	39	0.15	36	− 0.70	107	− 0.82	100	− 0.01	175	0.09	188
7	0.55	39	0.57	38	− 0.48	102	− 0.70	104	0.27	170	0.17	268
8	0.58	34	0.61	36	− 0.83	95	− 0.86	95	0.46	170	0.68	172
9	0.60	39	0.58	39	− 0.55	99	− 0.43	96	0.18	163	0.54	170
10	0.55	38	0.46	38	− 0.60	93	− 0.67	91	0.17	173	0.28	181
11	0.41	33	0.49	34	− 0.53	90	− 0.49	102	0.05	168	0.30	174
12	0.27	39	0.33	38	− 0.31	94	− 0.57	97	0.01	287	− 0.15	204
13	0.78	43	0.88	43	− 0.90	97	− 0.85	99	0.14	165	0.21	180
14	1.08	41	1.05	41	− 1.54	97	− 1.53	96	0.37	161	0.48	161
Wilcoxon signed rank test (all 14 subjects)												
* Δ*			0.01	− 1			− 0.02	1			0.16	5
* p*			0.715	0.368			0.808	0.938			0.007	0.326
Wilcoxon signed rank test (Subject 1, 6, 12 excluded)												
* Δ*			0.00	0			0.10	0			0.17	2
* p*			0.831	0.072			0.206	0.520			0.005	0.054

### The effect of flash-to-click delay on visual modulation of cAEPs

To examine the relationship between audiovisual temporal disparity and visual modulation of auditory processing, we sorted all the click stimuli by their flash-to-click delays (Fig. 1c). At first, we created 8 groups of with equal number of clicks in each group. In this case, the first click group was composed of clicks with a flash-to-click delay between 0 and 79 ms, while the last group was composed of clicks with a flash-to-click delay between 894 and 1731 ms. Detailed descriptive statistics on the flash-to-click delays were listed below (Table 2). Next, the cAEP waveforms were derived from each of the 8 click groups (Supplementary Fig. 1b), and therefore the contrast between the A and the AV-V conditions for each click group can represent for the cortical processing of click stimuli under the influence of visual modulation with a specific window of audiovisual temporal disparity.

**Table 2:** Descriptive statistics about the flash-to-click delays in each of the eight click groups.

Flash-to-click delay (ms)	Group								
	1	2	3	4	5	6	7	8	Rem.
Min	0	79	168	255	349	496	651	894	1856
Max	79	167	254	345	496	640	892	1731	1985
Range	79	88	87	90	146	144	241	837	129
Median	34	126	198	299	419	553	751	1051	1921
Count	46	46	46	46	46	46	46	46	2

We first compared the range of N1 amplitudes across the 8 click groups. It appeared that there was a larger range of N1 amplitude across the 8 click groups in the AV-V condition than the A condition (Supplementary Fig. 1c), although this difference was not statistically significant.

Next, one-way repeated-measure ANOVA was performed to test the statistical effect of click group on the change of N1 amplitude (amp_*N1*_) against the variance across subjects. We found a significant main effect of click group (*F*_10, 70_ = 2.72, *p* = 0.015 < 0.05). Given the small sample size, we also carried out a permutation test, where the correspondence between the click groups and the Δamp_*N1*_ were randomly scrambled for each subject independently. This allowed us to determine a false discovery rate of 1.0% when accepting 0.015 as the alpha level.

**Figure 3:**
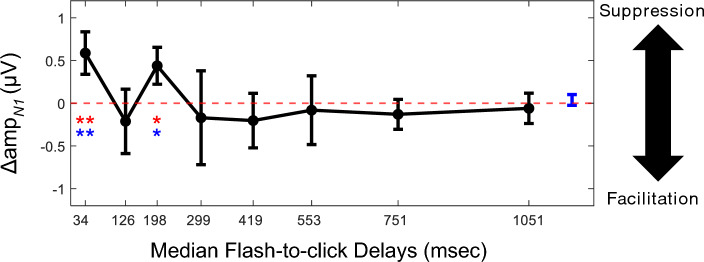
Effect of audiovisual temporal disparity on visual modulation of N1 amplitude. Median of change in N1 amplitude for each of the 8 click groups. The median of flash-to-click delays were used as horizontal coordinates. Errorbar, half of the inter-quartile range across subjects. The red-dash line, the null hypothesis with no visual modulation. Blue errorbar, the inter-quartile range of Δamp_*N1*_ across subjects without click grouping.

To further identify the specific click groups that demonstrated delay-dependent visual modulation, we performed Wilcoxon sign rank tests in each of the 8 click groups, comparing Δamp_*N1*_ with either 0 (i.e., assuming no visual modulation at all as the null hypothesis) or the Δamp_N1_ derived from each subject without click grouping (i.e., assuming no delay dependency as the null hypothesis). In both approaches, a significant suppression of N1 amplitudes, as indicated by a positive Δamp_*N1*_, was found for the 34-ms click group and the 198-ms group click group (Fig. 3). Again, we used the same permutation procedure described above to confirm that accepting both positive findings (34-ms: *p* = 0.008 < 0.01; 198-ms: *p* = 0.013 < 0.05) yielded an accumulated false discovery rate of 0.3% when Δamp_*N1*_ values were compared to zero. The other click groups failed to reveal a statistically significant visual modulation, suggesting that visual modulation in those ranges of audiovisual temporal disparity was less consistent across subjects. We also explored the other number (from 2 to 12) for click grouping and found that the pattern how visual modulation of N1 amplitude depends on audiovisual temporal disparity can be consistently observed using 7-bin, 8-bin, 9-bin, 10-bin, 11-bin grouping of clicks (Supplementary Figs. 2 and 3).

### Visual modulation of N1 amplitude predicted by audiovisual temporal disparity

**Figure 4:**
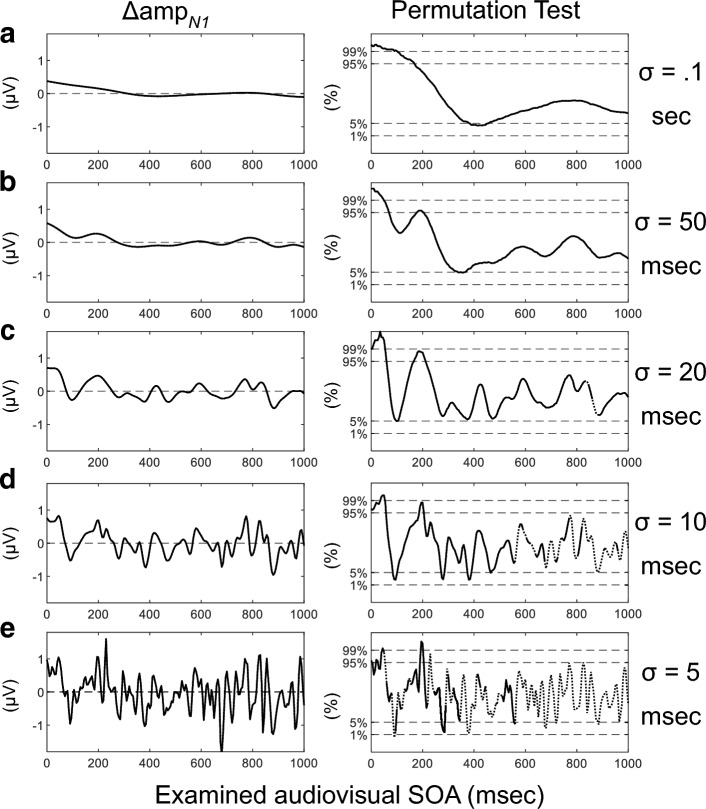
Visual modulation of N1 amplitude depends on audiovisual temporal disparity. (**a**–**e**) For kernels with different bandwidth (σ), change in N1 amplitude as predicted by audiovisual SOA derived from Gaussian-weight averaging of cAEPs. Left, the original Δamp_*N1*_. Right, proportion of permutation-derived Δamp_*N1*_ smaller than the original Δamp_*N1*_. Dotted line, peak detection with large variance indicated by latency beyond 150 ms or less than 55 ms.

Finally, we adopted from kernel regression procedure for weighing each of the 370 click epochs to predict the cAEP waveforms specific for a given audiovisual temporal disparity (audiovisual SOA), which we also termed as a Gaussian-weight averaging approach (Supplementary Fig. 4). For any given SOA, epochs were averaged with weight values derived from a Gaussian kernel centered at this SOA. The bandwidth of the Gaussian kernels was controlled by the parameter *σ*, which was selected to be 100-, 50-, 20-, 10-, 5-ms (Fig. 4a–e), concerning the trade-off between bias and variance of the prediction. Similarly, N1 amplitudes were measured and contrasted between the A and the AV-V conditions. The temporal course of visual modulation in N1 amplitude can be characterized by directly plotting Δamp_*N1*_ as a function of audiovisual SOA (Fig. 4a–e, Left).

The lack of clicks with long flash-to-click delays exerted additional variance to the prediction near the end of the evaluated SOA range. To alleviate its interference, we obtained the proportion of greater Δamp_*N1*_ than the data obtained through 1000 permutations, where all the flash-to-click delays were randomly assigned to the 370 click epochs (Fig. 4a–e, Right). Additionally, to monitor the quality of peak detection, N1 latency was measured at the same time.

Using the kernels with a large bandwidth (*σ* > 20 ms), we observed an overall transition from visual suppression to facilitation of N1 amplitude at ~ 300-ms SOA (Fig. 4a–c). Using the kernels with a smaller bandwidth, an early and transient facilitation can be identified at ~ 100-ms SOA (Fig. 4c–e). Such temporal dynamic was also partially captured by the analysis demonstrated earlier where the clicks were grouped in discrete bins. Furthermore, strong visual modulation on N1 amplitude was also revealed at multiple SOAs like 300- and 400-ms, when the kernels with a small bandwidth were used (Fig. 4d), suggesting multiple temporal integration windows for audiovisual interaction.

## Discussion

In this study, we examined and demonstrated the effect of audiovisual temporal disparity or stimulus onset asynchrony (SOA) on visual modulation of cortical auditory evoked potentials (cAEPs). The audiovisual interaction was investigated using similar approaches in two previous human ERP studies, with SOAs below 100 ms ^17^ and 70 ms ^16^, respectively. A few studies using extracellular recordings examined SOAs up to 500 ms in the superior colliculus ^1^ and 320 ms in auditory cortices ^34^. These studies have made the discoveries of the neural correlates to the “temporal window of integration” that were measured behaviorally, demonstrating strong evidence for a “coincidence detector” as a neurophysiological mechanism ^35–37^.

Long SOAs, despite not likely being involved with the temporal integration or temporal processing (perception of multisensory simultaneity and temporal order), are still possible for effective cross-modal modulation of sensory processing. This idea has been supported by both behavioral data ^24,38,39^ and some neurophysiological evidence ^23,40^. Lakatos et al. ^23^ pointed out that the optimal SOAs for tactile modulation of sound-evoked neuronal activities in their data were associated with the periodic intervals of several EEG oscillations. According to the “phase reset” hypothesis they proposed, a preceding tactile stimulus resets the phase of ongoing neural oscillations in the primary auditory cortex, which in turn determines the state of fluctuating auditory excitability. When the SOA between the preceding tactile stimulus and the following auditory stimulus is aligned to the high-excitability, up-phase of neural oscillation, the auditory stimulus evokes a larger response than when tactile-auditory SOA is aligned to the low-excitability, low-phase of neural oscillation. The observation of excitability fluctuation has been further evidenced with various behavioral and electrophysiological measurements, including extracellular recording ^34^, human ERP ^16^, phosphine induced by transcranial magnetic stimulation ^40,41^, and reaction time ^24,38,39^. Although our analysis was mainly focused on the prediction of visual modulation by audiovisual temporal disparity, the result did exhibit a pattern of fluctuating suppression/facilitation as SOA increased from 0 to 1000 ms. It is worth noting that neither auditory nor visual stimuli in this study was dedicated as a periodic inputs. Therefore, the oscillation in visual modulation we observed may reflect an intrinsic property of neural networks.

One of the many missions of the future multisensory research is to converge the knowledge established from extracellular recordings in animal models and from whole-brain imaging in humans. While data of intracranial recordings in human are still rare and challenging to obtain, scalp-EEG recordings from large animal models are quickly developing as a uniquely useful neurophysiological approach, such as marmoset ^42,43^ and cat ^44–48^.

Electrical and magnetic mappings of whole-brain activities during audiovisual perception have provided valuable insights on its neural mechanism involving intra-cortical functional connectivity^49^ and topographic re-distribution ^26^. Human auditory evoked potentials have been well-characterized for a variety of components as neural correlates to sound processing at different stages of ascending auditory pathway ^50,51^. The current study is the first scalp-recorded EEG multisensory study in animal models, and is, infrequently in literature, focused on auditory evoked potentials under visual modulation. We compared ERPs from the auditory-only condition with a derived condition by subtracting signal of the visual-only condition from the audiovisual condition, rather than compare the difference between audiovisual condition with a derived condition by “sum of the auditory and the visual conditions”. This allowed us to select peak components time-locked to auditory stimuli, which are supposed to have better interpretability for auditory processing.

To summarize, in this study we mainly characterized N1 amplitude in scalp-recorded auditory evoked potentials (AEPs) from cats under dexmedetomidine sedation as a measurement for visual modulation of auditory processing. We found that the delay function, sampled with both sparse grouping approach and fine-resolution weight-average approach, revealed a short-SOA effect peaking at ~ 100 ms, which was followed by a long-SOA effect characterizing the time course of visual modulation over ~ 1-s period. With the advantages of our animal models and experiment paradigms, future studies are expected to characterize the spectrotemporal features in normal and sensory-deprived subjects and to identify the neural mechanism underlying cross-modal interactions.

## Methods

All procedures were conducted in compliance with the National Research Council's Guide for the Care and Use of Laboratory Animals (8th edition; 2011), the Canadian Council on Animal Care's Guide to the Care and Use of Experimental Animals (1993), and the ARRIVE guidelines. Furthermore, the following procedures were also approved by Animal Care Committee (DOWB) for the Faculty of Medicine and Health Sciences at McGill University.

### Animal preparation and anesthesia protocol

Cats (*felis catus*) were obtained from a commercialized animal breeder for biomedical research (Marshall Bioresources). We recorded 14 cats with average age of 4.7 ± 1.5 years old, two of which were male. After subjects were sedated using dexmedetomidine (0.04 mg/kg, Dexdomitor, Zoetis) injected intramuscularly, the left eye was occluded using a black contact lens so that visual stimuli were presented unilaterally. Phenylephrine (Mydfrin, Alcon) was applied to the right eye to dilate the pupil, and saline drops were used as lubrication. Subjects were placed on a water-circulated heating pad (TP-400, Gaymar). Once vital signs (heart rate and SpO2) were stable, two 15-min recording sessions were carried out while the subject was breathing pure oxygen (Dispomed). At the end of the two recording sessions, data collection terminated in nine subjects and continued in the other five under isoflurane anesthesia for a separate study. Subject’s vital signs and electrode impedance were checked between the two sessions. At the end of data collection, electrodes and contact lens were removed before atipamezole (Antisedan, Zoetis) was administrated intramuscularly to facilitate recovery from the dexmedetomidine sedation.

### Visual and auditory stimuli

The visual stimuli consisted of flashes that were presented to subjects from a 5-mm-diameter light-emitting diode (~ 11 degrees of visual field, LED, DigiKey). The intensity of flash stimuli was calibrated to 10 cd/m^2^ by adjusting the voltage magnitude of a 300-us-long squared pulse as the input signal to the LED. The auditory stimuli were 300- μs-long clicks emitted by an 8-cm-diamter loudspeaker (Fostex). The sound level of the click stimuli was calibrated to 55 dB SPL using a sound meter (Model 2250, B&K). Both auditory and visual stimulation signals were generated by the same digital-to-analogue processor (RZ2, TDT). The LED was attached to the top of the loudspeaker and placed 8-cm away from the subject at the direction of 45 degrees right to the midline.

To manipulate the timing of auditory and visual stimulus, two independent, 57-s-long pulse trains for triggering clicks and flashes, respectively, were pre-made in Matlab using a Poisson random process and loaded into the stimulus/recording software (Synapse, TDT). The auditory stimulus train contained 370 click pulses and the visual stimulus train contained 70 flash pulses. The minimal inter-click interval was set to 20 ms and the minimal inter-flash interval was set to 500 ms. The auditory and the visual stimulus trains always started and stopped simultaneously in each session. Auditory only (A), visual only (V), and audiovisual (AV) stimulus trains were played alternatively in order and repeated for 10 times.

Since click train and flash train were “out of sync”, a flash-to-click delay can be determined for each of the 370 clicks as the retrospective interval between the click onset and the onset of the immediately preceding flash (Supplementary Fig. 5). The flash-to-click delay spanned from 0 to beyond 1000 ms, although it does not conform to a uniform distribution.

### EEG recording and signal processing

Three 25G stainless steel needles were placed subdermal as recording electrodes (Supplementary Fig. 6). The active electrode was placed near the midpoint of subject’s interaural line, while the reference electrode was placed below the right ear (ipsilateral to the side of visual stimulation). The ground electrode was placed on the subject’s dorsum (~ 10 cm behind shoulder blade near the midline). The impedance of both active and reference electrodes was maintained below 3 kΩ during recording. The signal was amplified and digitized with a pre-amplifier (Medusa4Z, TDT), streamed onto a digital signal processor (RZ2, TDT), and stored on a computer hard drive. The analogue signal was digitized at a sample rate of ~ 6.1 kHz and passed through an anti-aliasing filter between 0.1 Hz and 1830 Hz.

All data analysis was performed offline. Signal was digitally notched at 60 Hz before passing through a band-pass filter (1–10 Hz) for cortical auditory evoked potentials. Then, the filtered signals from the same stimulus conditions were averaged. For AV-V condition, AEPs were derived from subtracting visual only (V) session average from audiovisual (AV) session average. For A condition, AEPs was derived from auditory only (A) session average.

Flash-to-click lags were calculated for each individual click as the delay of its onset to the onset of its preceding flash for audiovisual stimulus. Epochs were extracted between 200-ms pre-click and 400-ms post-click.

In time-binned sub-group averaging, epochs were ordered ascendingly by flash-to-click lags. Taking 8-bin grouping as an example, bins were created for every 46 epochs and labeled as the median flash-to-click lags. The first 368 epochs were included, with the remaining 2 epochs discarded. In Gaussian-weight averaging, SOAs were selected from 0- to 1000-ms with a 5-ms step. For each SOA, a Gaussian kernel function with one of the five bandwidths (σ = 5, 10, 20, 50, 100 ms) was centered at the SOA. Clicks within ± 3 σ range were included into the average with weight values given by the Gaussian kernel functions. Click epochs with flash-to-click lags more deviating away from the SOA (i.e., the peak of Gaussian kernel) therefore contributed less to the averaged waveform.

### Extraction of N1 amplitude

First, a peak latency of N1 was determined from all click responses averaged together, which was then used as a reference. To find the peak of N1 in cAEP waveforms derived from sub-groups of clicks, we customized a Matlab program that identified all local minima on each waveform and selected the minima with the closest latency to the reference latency previously determined. The amplitude of N1 was measured in reference to the baseline (from 5-ms before to 5-ms after the click onsets).

### Statistics

Repeated-measure ANOVA and Wilcoxon sign rank test were performed on Matlab using Statistics and Machine Learning Toolbox™. Permutation tests were performed using customized Matlab programs. For Gaussian-weight, N1 amplitudes were derived using the grand average across subjects. To test for statistical significance, 1000 permutations were performed by randomizing the mapping between the epochs and their flash-to-click delays.

### Supplementary Information

Supplementary Figures.

## Data Availability

The datasets generated during and/or analysed during the current study are available from the corresponding author on reasonable request.

## References

[1] Meredith MA, Nemitz JW, Stein BE (1987). Determinants of multisensory integration in superior colliculus neurons. I. Temporal factors. J. Neurosci..

[2] Koelewijn T, Bronkhorst A, Theeuwes J (2010). Attention and the multiple stages of multisensory integration: A review of audiovisual studies. Acta Psychol. (Amst.).

[3] Dixon NF, Spitz L (1980). The detection of auditory visual desynchrony. Perception.

[4] Fister JK, Stevenson RA, Nidiffer AR, Barnett ZP, Wallace MT (2016). Stimulus intensity modulates multisensory temporal processing. Neuropsychologia.

[5] Stevenson RA (2014). Multisensory temporal integration in autism spectrum disorders. J. Neurosci..

[6] Hirsh IJ, Sherrick CE (1961). Perceived order in different sense modalities. J. Exp. Psychol..

[7] Vatakis A, Navarra J, Soto-Faraco S, Spence C (2008). Audiovisual temporal adaptation of speech: Temporal order versus simultaneity judgments. Exp. Brain Res..

[8] van Wassenhove V, Grant KW, Poeppel D (2007). Temporal window of integration in auditory-visual speech perception. Neuropsychologia.

[9] Harrar V, Harris LR, Spence C (2017). Multisensory integration is independent of perceived simultaneity. Exp. Brain Res..

[10] Chen Y-C, Spence C (2013). The time-course of the cross-modal semantic modulation of visual picture processing by naturalistic sounds and spoken words. Multisens. Res..

[11] Chen Y-C, Spence C (2018). Audiovisual semantic interactions between linguistic and nonlinguistic stimuli: The time-courses and categorical specificity. J. Exp. Psychol. Hum. Percept. Perform..

[12] Chen Y-C, Spence C (2018). Dissociating the time courses of the cross-modal semantic priming effects elicited by naturalistic sounds and spoken words. Psychon. B Rev..

[13] Schormans AL (2017). Audiovisual temporal processing and synchrony perception in the rat. Front. Behav. Neurosci..

[14] Stevenson RA, Wallace MT (2013). Multisensory temporal integration: Task and stimulus dependencies. Exp. Brain Res..

[15] Stevenson RA, Zemtsov RK, Wallace MT (2012). Individual differences in the multisensory temporal binding window predict susceptibility to audiovisual illusions. J. Exp. Psychol. Hum. Percept. Perform..

[16] Naue N (2011). Auditory event-related response in visual cortex modulates subsequent visual responses in humans. J. Neurosci..

[17] Thorne JD, De Vos M, Viola FC, Debener S (2011). Cross-modal phase reset predicts auditory task performance in humans. J. Neurosci..

[18] Lu Y, Paraskevopoulos E, Herholz SC, Kuchenbuch A, Pantev C (2014). Temporal processing of audiovisual stimuli is enhanced in musicians: Evidence from magnetoencephalography (MEG). PLoS One.

[19] Liu B, Jin Z, Wang Z, Gong C (2011). The influence of temporal asynchrony on multisensory integration in the processing of asynchronous audio-visual stimuli of real-world events: An event-related potential study. Neuroscience.

[20] Franciotti R, Brancucci A, Della Penna S, Onofrj M, Tommasi L (2011). Neuromagnetic responses reveal the cortical timing of audiovisual synchrony. Neuroscience.

[21] Basharat A, Adams MS, Staines WR, Barnett-Cowan M (2018). Simultaneity and temporal order judgments are coded differently and change with age: An event-related potential study. Front. Integr. Neurosci..

[22] Oray S, Lu Z-L, Dawson ME (2002). Modification of sudden onset auditory ERP by involuntary attention to visual stimuli. Int. J. Psychophysiol..

[23] Lakatos P, Chen C-M, O'Connell MN, Mills A, Schroeder CE (2007). Neuronal oscillations and multisensory interaction in primary auditory cortex. Neuron.

[24] Fiebelkorn IC (2011). Ready, set, reset: Stimulus-locked periodicity in behavioral performance demonstrates the consequences of cross-sensory phase reset. J. Neurosci..

[25] Sauseng P (2007). Are event-related potential components generated by phase resetting of brain oscillations? A critical discussion. Neuroscience.

[26] Cappe C, Thut G, Romei V, Murray MM (2010). Auditory–visual multisensory interactions in humans: Timing, topography, directionality, and sources. J. Neurosci..

[27] Giard MH, Peronnet F (1999). Auditory-visual integration during multimodal object recognition in humans: A behavioral and electrophysiological study. J. Cogn. Neurosci..

[28] Molholm S (2002). Multisensory auditory–visual interactions during early sensory processing in humans: A high-density electrical mapping study. Brain Res. Cogn. Brain Res..

[29] Fort A, Delpuech C, Pernier J, Giard M-H (2002). Early auditory–visual interactions in human cortex during nonredundant target identification. Brain Res. Cogn. Brain Res..

[30] Vidal J, Giard M-H, Roux S, Barthélémy C, Bruneau N (2008). Cross-modal processing of auditory–visual stimuli in a no-task paradigm: A topographic event-related potential study. Clin. Neurophysiol..

[31] Cappe C, Thelen A, Romei V, Thut G, Murray MM (2012). Looming signals reveal synergistic principles of multisensory integration. J. Neurosci..

[32] Mercier MR, Cappe C (2020). The interplay between multisensory integration and perceptual decision making. NeuroImage.

[33] Caron-Desrochers L, Schönwiesner M, Focke K, Lehmann A (2018). Assessing visual modulation along the human subcortical auditory pathway. Neurosci. Lett..

[34] Kayser C, Petkov CI, Logothetis NK (2008). Visual modulation of neurons in auditory cortex. Cereb. Cortex.

[35] Leone LM, McCourt ME (2013). The roles of physical and physiological simultaneity in audiovisual multisensory facilitation. i-Perception.

[36] Rowland BA, Stanford TR, Stein BE (2007). A model of the neural mechanisms underlying multisensory integration in the superior colliculus. Perception.

[37] Truszkowski TL (2017). A cellular mechanism for inverse effectiveness in multisensory integration. Elife.

[38] Diederich A, Schomburg A, Colonius H (2012). Saccadic reaction times to audiovisual stimuli show effects of oscillatory phase reset. PLoS One.

[39] Diederich A, Schomburg A, Van Vugt M (2014). Fronto-central theta oscillations are related to oscillations in saccadic response times (SRT): An EEG and behavioral data analysis. PLoS One.

[40] Romei V, Gross J, Thut G (2012). Sounds reset rhythms of visual cortex and corresponding human visual perception. Curr. Biol..

[41] Romei V, Murray MM, Cappe C, Thut G (2013). The contributions of sensory dominance and attentional bias to cross-modal enhancement of visual cortex excitability. J. Cogn. Neurosci..

[42] Itoh K, Iwaoki H, Konoike N, Igarashi H, Nakamura K (2021). Noninvasive scalp recording of the middle latency responses and cortical auditory evoked potentials in the alert common marmoset. Hearing Res..

[43] Itoh K, Nejime M, Konoike N, Nakada T, Nakamura K (2015). Noninvasive scalp recording of cortical auditory evoked potentials in the alert macaque monkey. Hearing Res..

[44] Presacco A, Middlebrooks JC (2018). Tone-evoked acoustic change complex (ACC) recorded in a sedated animal model. J. Assoc. Res. Oto..

[45] Presacco A, Middlebrooks JC (2018). Tone-evoked acoustic change complex (ACC) in an animal model. J. Acoust. Soc. Am..

[46] Richardson ML (2022). Temporal pitch sensitivity in an animal model: Psychophysics and scalp recordings: Temporal pitch sensitivity in cat. J. Assoc. Res. Oto..

[47] Heidari M (2019). Evoked potentials as a biomarker of remyelination. Proc. Natl. Acad. Sci. USA.

[48] Mitzelfelt T, Bao X, Barnes P, Lomber SG (2023). Visually evoked potentials (VEPs) across the visual field in hearing and deaf cats. Front. Neurosci..

[49] Keitel A, Ince RA, Gross J, Kayser C (2017). Auditory cortical delta-entrainment interacts with oscillatory power in multiple fronto-parietal networks. NeuroImage.

[50] Eggermont JJ, Ponton CW (2002). The neurophysiology of auditory perception: From single units to evoked potentials. Audiol. Neurootol..

[51] Scherg M, Vajsar J, Picton TW (1989). A source analysis of the late human auditory evoked potentials. J. Cogn. Neurosci..

