# Supplementary material for: Visual modulation of auditory evoked potentials in the cat
Source: Sci Rep. 2024 Mar 26;14:7177. doi: 10.1038/s41598-024-57075-1 (PMC10965913; doi:10.1038/s41598-024-57075-1)
Supplement: Supplementary file 1 — Supplementary Figures. [file 41598_2024_57075_MOESM1_ESM.pdf]

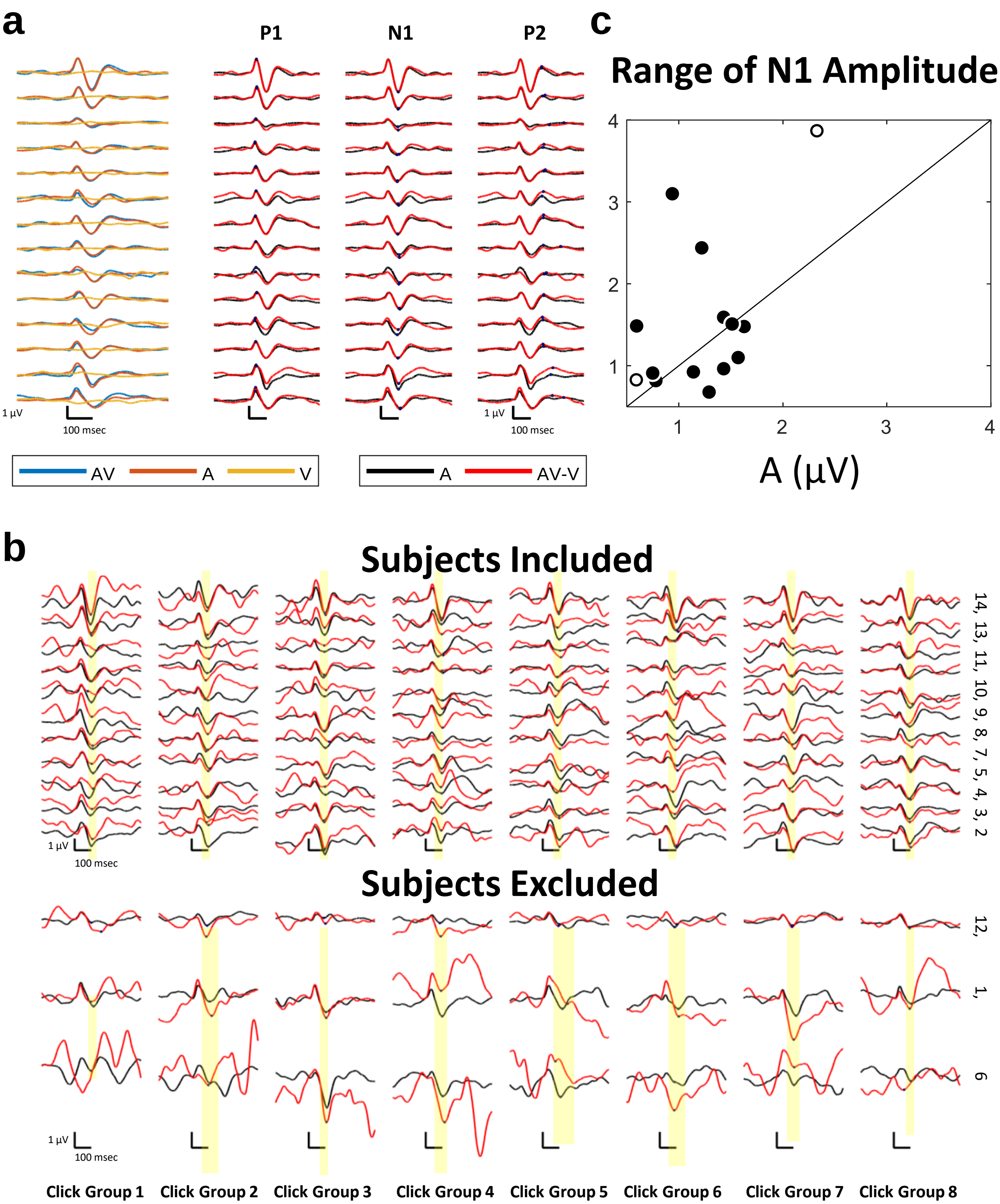

Supplementary Figure 1. cAEP waveforms in individual subjects and variation in N1 amplitude. **a)** Epochs time-locked to click onsets averaged in each of the 14 cat subjects. Left, cAEPs derived from the three original stimulus conditions. Right, contrast of cAEPs between the original A condition and the derived AV-V condition. Blue dots, identified peaks in P1-N1-P2 complex. **b)** Contrast of cAEP waveforms for each of the 8 click groups in individual subjects. Note that subjects excluded exhibited more noise in the waveform that made P1 identification more difficult (e.g., Click Group 1 in Subject 12). **c)** Range of N1 amplitude across the click groups compared between the A and the AV-V. conditions.

Supplementary Figure 1

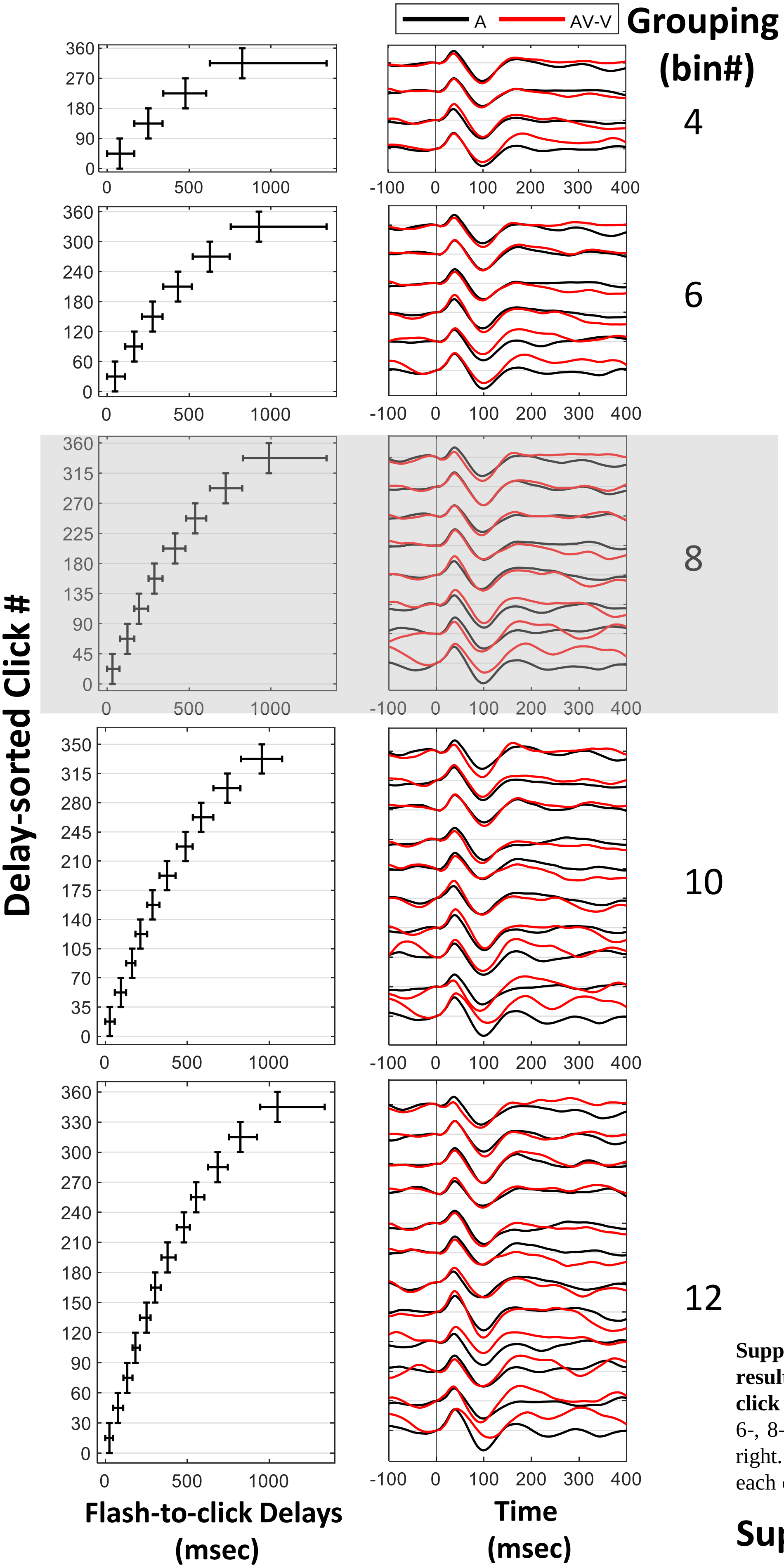

Supplementary Figure 2. Exploratory results using different number of bins in click grouping. cAEP waveforms with 4-, 6-, 8-, 10, and 12-bin are illustrated on the right. The ranges of flash-to-click delays in each click bin are shown on the left.

Supplementary Figure 2

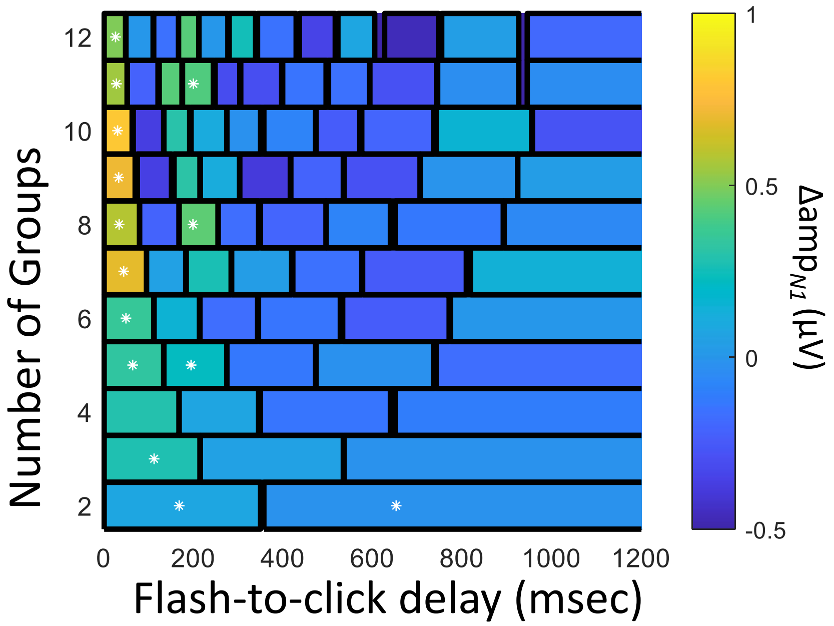

N-bin Grouping	Group No.	Flash-to-click Delays (msec)			vs. Δ w/o grouping		vs. Δ = 0	
		Median	Min	Max	p-Value	FDR	p-Value	FDR
2	1	168	0	349	**0.041**	**0.021**	**0.021**	**0.007**
2	2	653	357	1985	**0.016**	**0.004**	0.424	0.274
3	1	112	0	214	**0.016**	**0.006**	**0.016**	**0.011**
4	1	79	0	167	0.062	**0.027**	**0.041**	**0.014**
5	1	64	0	133	**0.041**	**0.017**	0.062	**0.031**
5	2	194	134	274	**0.041**	**0.033**	**0.013**	**0.004**
6	1	49	0	111	**0.041**	**0.018**	0.050	**0.014**
7	1	44	0	95	**0.016**	**0.005**	**0.016**	**0.004**
8	1	34	0	79	**0.008**	**0.003**	**0.008**	**0.001**
8	3	198	168	254	**0.026**	**0.016**	**0.013**	**0.004**
9	1	33	0	69	**0.008**	**0.002**	**0.008**	**0.001**
9	3	181	156	214	0.091	0.062	0.091	**0.036**
10	1	30	0	63	**0.008**	**0.001**	**0.010**	**0.001**
10	4	240	194	274	0.328	0.477	0.110	**0.046**
11	1	28	0	51	**0.016**	**0.006**	**0.008**	**0.002**
11	4	199	175	244	**0.016**	**0.011**	**0.013**	**0.004**
12	1	26	0	48	**0.041**	**0.017**	**0.016**	**0.004**
12	4	184	166	212	0.062	**0.046**	**0.021**	**0.009**

Supplementary Figure 3. Exploratory results on N1 amplitude using different number of bins in click grouping. N1 amplitudes were measured and compared between the A and the AV-V conditions. Median of Δamp$_{N1}$ across subjects are color coded. Significance marked as * and listed in the table below.

Supplementary Figure 3

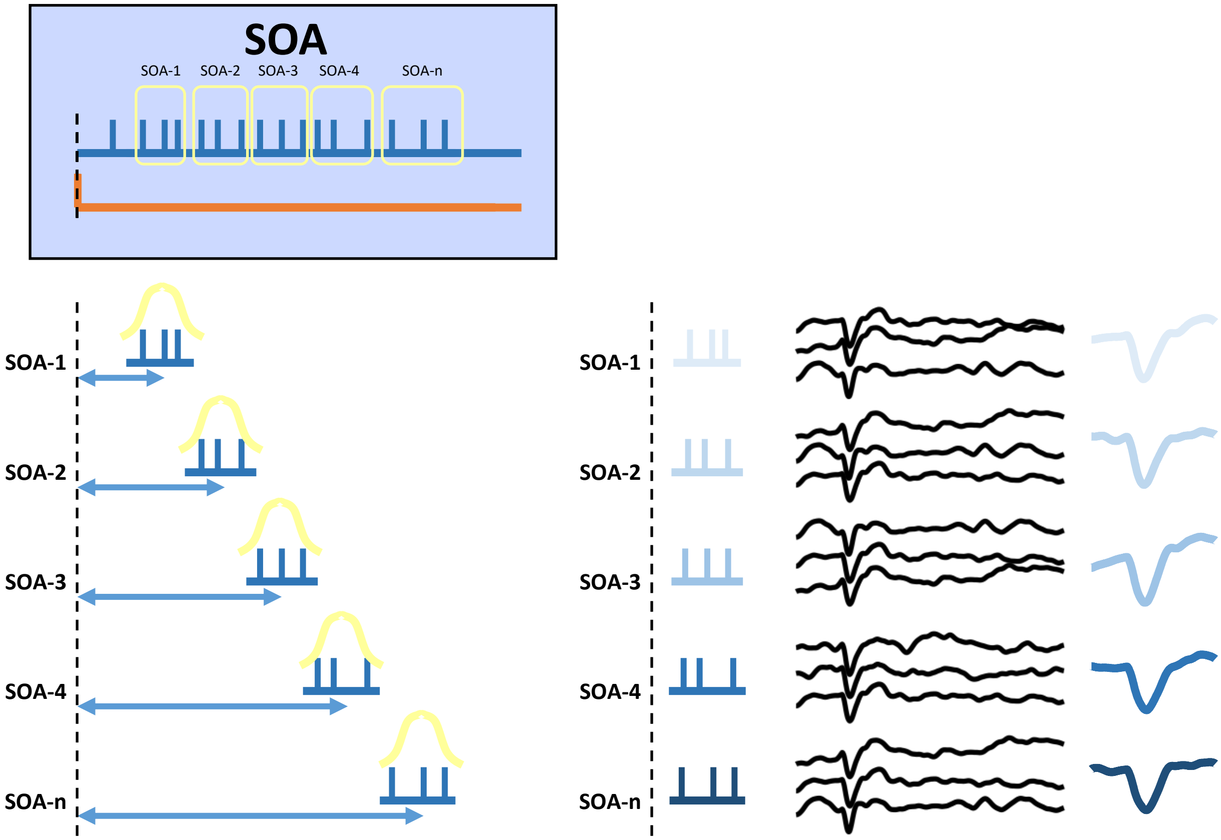

Supplementary Figure 4. Illustration of Gaussian-weight averaging approach. Left, similar as in kernel regression, epochs time-locked to clicks were sorted by flash-to-click delays and were assigned weight by a Gaussian kernel for the prediction of each audiovisual temporal disparity (SOA). Right, for a given SOA, N1 amplitude was measured from cAEP waveform obtained from the weight-average of epochs before the difference was taken between the A and the AV-V conditions.

Supplementary Figure 4

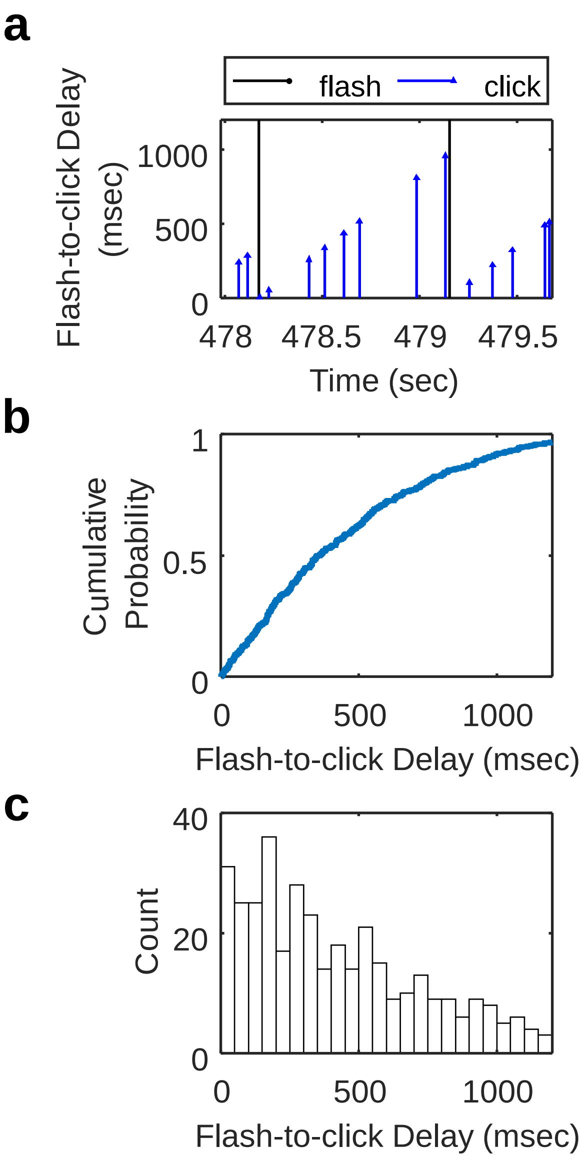

Supplementary Figure 5. Distribution of flash-to-click delays. a) An exemplar 1.5-second interval of audiovisual stimulus train. Flash and click onsets were illustrated as in the stem plot. For clicks, the stem height indicates the retrospective interval to its immediately preceding flash. **b)** Cumulative probability function of flash-to-click delays estimated from the 370 clicks in the audiovisual stimulus train in total. **c)** Histogram of the flash-to-click delays using 50-msec bin-width.

Supplementary Figure 5

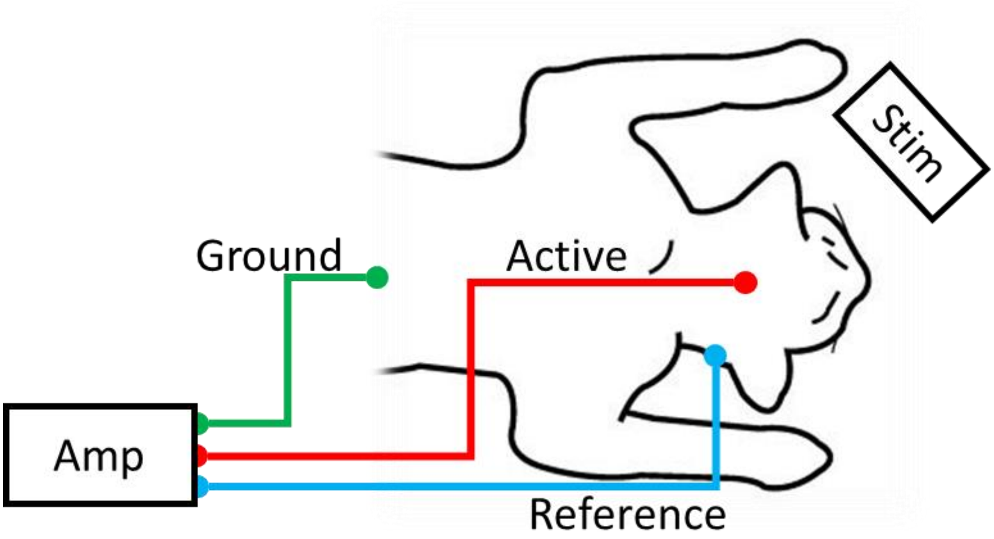

Supplementary Figure 6. Placement of subdermal needles as EEG electrodes.
